# Everolimus-Eluting versus Paclitaxel-Eluting Stents in Percutaneous Coronary Intervention: Meta-Analysis of Randomized Trials

**DOI:** 10.1155/2012/126369

**Published:** 2012-05-09

**Authors:** Ashraf Alazzoni, Ayman Al-Saleh, Sanjit S. Jolly

**Affiliations:** Departments of Medine and Cardiology, Hamilton Health Sciences, McMaster University, Hamilton, ON, Canada L8L 2X2

## Abstract

*Background*. Individual randomized trials have suggested that everolimus-eluting stents may have improved clinical outcomes compared to paclitaxel-eluting stents, but individual trials are underpowered to examine outcomes such as mortality and very late stent thrombosis. *Methods*. Medline, Cochrane, and conference proceedings were searched for randomized trials comparing everolimus versus paclitaxel-eluting stents for percutaneous coronary intervention. *Results*. 6792 patients were included from 4 randomized controlled trials. Stent thrombosis was reduced with everolimus stents versus paclitaxel stents (0.7% versus 2.3%; OR: 0.32; CI: 0.20–0.51; *P* < 0.00001). The reductions in stent thrombosis were observed in (i) early stent thrombosis (within 30 days) (0.2% versus 0.9%; OR: 0.24; *P* = 0.0005), (ii) late (day 31–365) (0.2% versus 0.6%; OR: 0.32; *P* = 0.01), and (iii) very late stent thrombosis (>365 days) (0.2% versus 0.8%; OR: 0.34; *P* = 0.009). The rates of cardiovascular mortality were 1.2% in everolimus group and 1.6% in paclitaxel group (OR: 0.85; *P* = 0.43). Patients receiving everolimus-eluting stents had significantly lower myocardial infarction events and target vessel revascularization as compared to paclitaxel-eluting stents. *Interpretation*. Everolimus compared to paclitaxel-eluting stents reduced the incidence of early, late, and very late stent thrombosis as well as target vessel revascularization.

## 1. Introduction

Bare metal stents (BMSs) were introduced to improve the acute results of coronary angioplasty and to prevent restenosis compared to balloon angioplasty [1]. The first generation of drug eluting stents (DESs) demonstrated significant reductions in restenosis and target vessel revascularization (TVR) compared with BMS [2]. However, meta-analyses of randomized trials have suggested an excess in late stent thrombosis (ST) for paclitaxel and sirolimus DES compared to BMS [3].

 The first Paclitaxel-eluting stent (PES), used widely, was the Taxus Express stent (Boston Scientific, Natick, MA, USA), and this stent was tested against BMS in multiple randomized trials and demonstrated nearly 50% reduction in target lesion revascularization (TLR) and TVR. However, there was an excess of very late ST (>1 year) in those patients who were treated with PES compared to BMS [1]. Subsequently, a 2nd PES stent has been approved, the Taxus Libert stenté, (Boston Scientific, Natick, MA, USA) which had the same drug and polymer but had thinner struts and improved deliverability. The TAXUS ATLAS study [4] compared outcomes with Taxus Liberte to historical controls treated with Taxus Express and showed similar outcomes.

 The 2nd generation DES, the Xience V Everolimus-eluting stent (EES) (Abbot Vascular, Santa Clara, CA, USA) consists of the multilink vision cobalt chromium platform with a nonerodible polymer and everolimus, a synthetic derivative of Sirolimus. Individual randomized trials have suggested reduced rates of myocardial infarction (MI) and early ST with the EES versus PES [5–8]. However, what remains unanswered is if the newer generation Everolimus-eluting stents reduce the rate of late and very late ST as well as mortality. A meta-analysis of available trials may allow increased power for important clinical outcomes that individual trials are not powered to compare such as mortality and very late ST. 

The objective of this meta-analysis was to compare the efficacy and safety of EES versus PES especially with regards to the patient important outcomes of ST (early, late, and very late), cardiovascular death and MI.

## 2. Methods

### 2.1. Criteria for Study Selection

We selected randomized controlled trials that compared the use of EES and PES in percutaneous coronary intervention (PCI).

### 2.2. Outcomes and Definitions

The primary outcomes of interest was ST, subclassified as early (within 30 days), late (31–365 days), and very late (>365 days) and cardiovascular death. Other outcomes included MI and TVR. ST was adjudicated according to the criteria for definite or probable ST of the Academic Research Consortium [9]. MI was defined as a typical rise and fall in concentrations of troponin or creatinine kinase-MB with at least one of the following: ischemic symptoms, development of pathological Q waves, ischemic electrocardiographic changes, or pathological findings of an acute MI in one trial [6]. Also, it was defined as either as the development of new pathologic Q waves 0.4 seconds or longer in duration in 2 or more contiguous leads or as an elevation of creatinine phosphokinase levels to more than 2 times normal with positive levels of creatinine phosphokinase MB in one trial [7], while it was defined as an elevation of CK to ≥2 times the upper limit of normal with elevated CK-MB in the absence or presence of new pathological Q waves on the electrocardiogram (non-Q- and Q-wave MI, resp.) in two trials [5, 8].

A composite of safety and efficacy (all-cause mortality, MI, and TVR) was the primary end point in one trial [6], while the composite of cardiac death, MI, or ischemia-driven TVR was the primary end point in 3 trials [5, 7, 8]. ST was one of the secondary end points in all trials.

In the three trials [6–8], at least 300 mg of Aspirin was administered before catheterization as well as a ≥300 mg oral dose of clopidogrel was recommended before the procedure. In the fourth trial, the periprocedural pharmaceutical treatment was administrated according to standard hospital practice without specification [5]. Maintenance therapy with Clopidogrel consisted of a daily dose of 75 mg for at least 6 months in two trials [5, 7] and at least 12 months in the two other trials [6, 8]. Maintenance therapy with Aspirin consisted of a daily dose of ≥75 mg for at least 1 year in one trial [5] while ≥80 mg indefinitely in two trials [7, 8]. In the fourth trial, patients were maintained on 80 mg of aspirin indefinitely [6]. There was no significant difference between both groups regarding compliance with antiplatelet therapy up to 1 year followup.

All trials reported the clinical outcomes of interest from 24-month up to a 4-year follow-up period (Table 1). Routine follow-up angiography was part of the study protocol in two trials at 180 days and 8 months, respectively [5, 7]. No routine follow-up angiography was planned in the other two trials [6, 8]. Each trial specified the stent platform that they used (Table 2).

### 2.3. Data Sources

We searched PubMed and the Cochrane Library for randomized controlled trials comparing EES with the PES in PCI. We limited our search to only the publications in English language. In addition, we manually searched the abstracts submitted to the American College of Cardiology (ACC), the American Heart Association (AHA), the European Society of Cardiology (ESC), and Transcatheter Therapeutics (TCT) up to May 27, 2011 (Diagram 1). Also, we contacted trials' authors for further data as needed.

### 2.4. Data Collection and Assessment of Quality

Studies were selected, and data were extracted independently by 2 reviewers (A. Alazzoni and A. Al-Saleh). Disagreements were resolved by consensus. We recorded the following clinical and angiographic characteristics, in addition to the number of participating patients: age, sex, diabetes mellitus, hypertension, hyperlipidemia, number of target lesions, location of target lesion, reference-vessel diameter, minimal luminal diameter, diameter stenosis, and lesion length. Duration of clinical followup and whether or not a followup using angiography was done as well as duration of aspirin and Thienopyridine use were also recorded. All available data was utilized including full publications, abstracts, and online late breaking presentations provided by the principal investigators.

We evaluated the quality of the involved trials using the Cochrane Collaboration's tool for assessing risk of bias [10]. In a similar manner to data collection, trials were evaluated independently by 2 reviewers (A. Alazzoni and A. Al-Saleh). Disagreements were resolved by consensus.

### 2.5. Statistical Analysis

All analyses were performed based on intention-to-treat data. Odds ratios (ORs) with 95% confidence intervals (CIs) were computed as summary statistics. The pooled OR was calculated with the Mantel-Haenszel method for fixed effects [11] and for a sensitivity analysis with the Mantel-Haenszel method for random effects. To assess heterogeneity across trials, we used the Cochran *Chi^2^* test based on the pooled OR by Mantel-Haenszel. Heterogeneity was also assessed by means of statistic as proposed by Higgins et al. [12]. Results were considered statistically significant at *P* ≤ 0.05. There was no adjustment for multiple comparisons. Statistical analyses were performed with Review Manager (RevMan) software version 5.

## 3. Results

**Figure 1:**
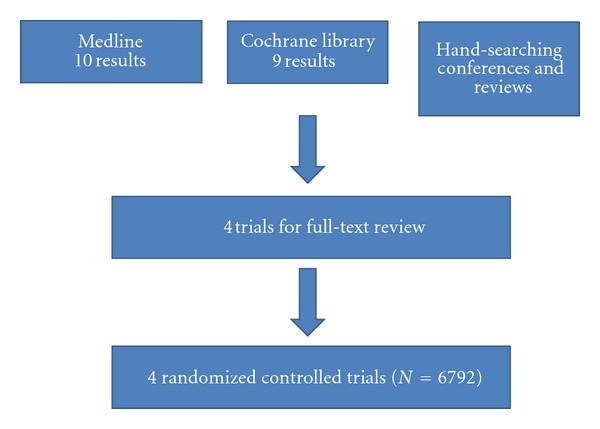
Data source flow chart diagram.

Our search identified 4 randomized controlled trials (Figure 1) that compared EESs and PESs use in 6792 patients during PCI [5–8]. Also, updates regarding all the trials were found through our manual search of PubMed [13–16]. Follow-up duration was 24 months in two trials [14, 16], 36 months in one trial [15], and 48 months in one trial [13] (Table 1). The abstracted data represents the longest available follow-up data.

 Overall, the number of patients treated with EES was 4247, while the control arm had 2541 patients who were treated with PES (Table 1). The mean age of patients is 62.9 in both groups. There were no significant differences between patients treated with the EESs and the PESs regarding the rate of diabetes. The proportion of patients with diabetes ranged from 17.1% to 32% in the EES group compared with 19% to 32.5% in the PES group. Only one trial allowed enrollment of patients with STEMI or recent MI [6]. Three trials excluded patients with vessel diameters <2.5 mm [5, 7, 8]. Similarly, patients assigned to the two drug-eluting stent types did not differ with respect to the main angiographic characteristics of the lesions.

### 3.1. ST

**Figure 2:**
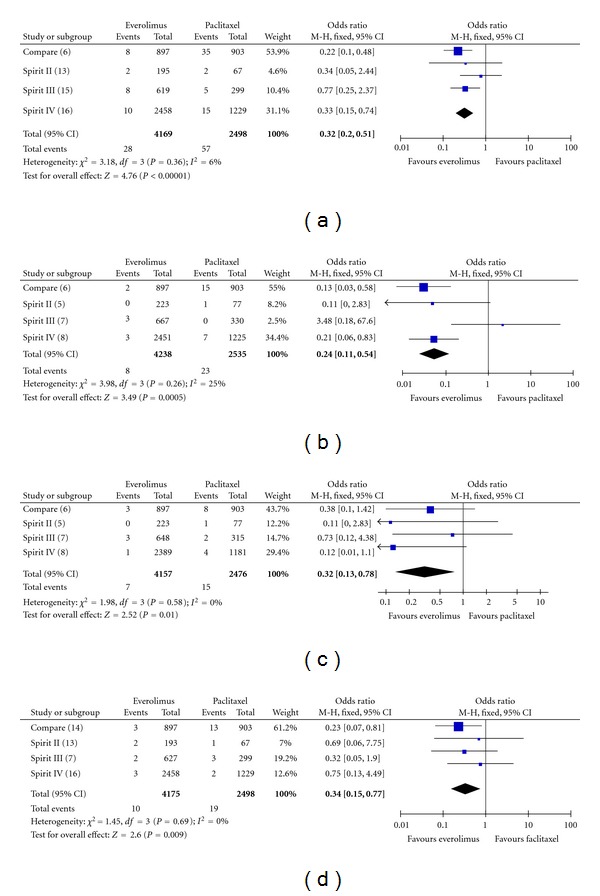
Odds ratio of stent thrombosis ((a): all, (b): early, (c): late, and (d): very late stent thrombosis) associated with everolimus-eluting stent versus paclitaxel-eluting stent.

The incidence of ST (definite and probable) at all followups was 0.7% (28 of 4169) among patients treated with the EES and 2.3% (57 of 2498) among patients treated with the PES (OR: 0.32, 95% CI: 0.20–0.51; *P* < 0.00001), with no significant study heterogeneity (Chi^2^ = 3.18; *P* = 0.36; *I* ^2^ = 6%) (Figure 2(a)). For definite ST, the incidence at all followups was 0.5% (21 of 4179) among patients treated with the EES and 1.6% (40 of 2499) among patients treated with the PES (OR: 0.33, 95% CI: 0.19–0.57; *P* = 0.0001).

 Looking specifically at early ST (0–30 days), the incidence was 0.2% (8 of 4238) among patients treated with the EES and 0.9% (23 of 2535) among patients treated with the PES (OR: 0.24, 95% CI: 0.11–0.54; *P* = 0.0005), with no significant study heterogeneity (Chi^2^ = 3.98; *P* = 0.26; *I* ^2^ = 25%) (Figure 2(b)).

Furthermore, the incidence of late ST (31–365 days) was 0.2% (7 of 4157) among patients treated with the EES and 0.6% (15 of 2476) among patients treated with the PES (OR: 0.32, 95% CI: 0.13–0.78; *P* = 0.01), with no significant study heterogeneity (Chi^2^ = 1.98; *P* = 0.58; *I* ^2^ = 0%) (Figure 2(c)). Regarding very late ST (>365 days), the incidence at all followups was 0.2% (10 of 4175) among patients treated with the EES and 0.8% (19 of 2498) among patients treated with the PES (OR: 0.34, 95% CI: 0.15–0.77; *P* = 0.009), with no significant study heterogeneity (Chi^2^ = 1.45; *P* = 0.69, *I* ^2^ = 0%) (Figure 2(d)).

### 3.2. Death

**Figure 3:**
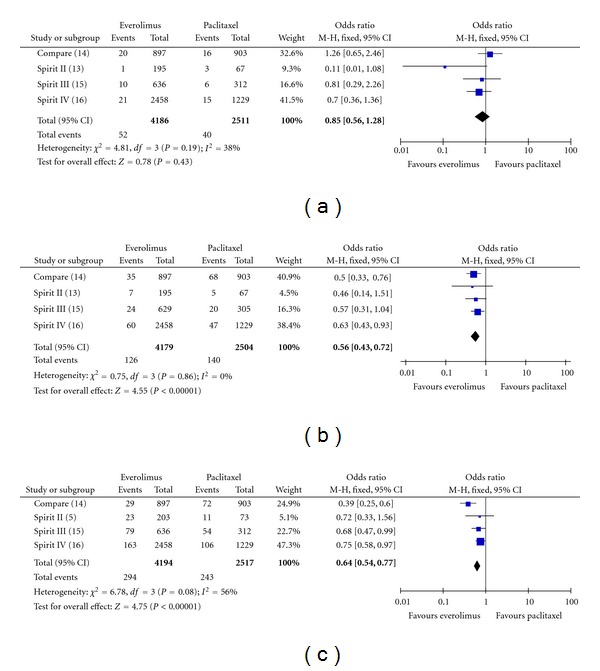
Odds ratio of (a): cardiac death (b): myocardial infarction and (c): target vessel revascularization associated with everolimus-eluting stent versus paclitaxel-eluting stent.

The incidence of all-cause death at all followups was 2.5% (106 of 4194) among patients treated with the EES and 3.2% (80 of 2517) among patients treated with the PES (OR: 0.8, 95%; CI: 0.59–1.07; *P* = 0.14). The incidence of cardiovascular death at all followup was 1.2% (52 of 4186) among patients treated with the EES and 1.6% (40 of 2511) among patients treated with the PES (OR: 0.85, 95% CI: 0.56–1.28; *P* = 0.43), with no significant study heterogeneity (Chi^2^ = 4.81; *P* = 0.19; *I* ^2^ = 38%) (Figure 3(a)).

### 3.3. MI

EESs were significantly more effective in the reduction of MI (Figure 3(b)). The incidence of MI at all followup was 3.0% (126 of 4179) among patients treated with the EES and 5.6% (140 of 2504) among patients treated with the PES (OR: 0.56; 95% CI: 0.43–0.72; *P* < 0.00001).

### 3.4. TLR (Ischemia-Driven) and TVR

Regarding ischemia-driven TLR, EESs were significantly more effective compared to PES with an incidence at all followups of 4.2% compared to 6.8% (OR: 0.57; CI: 0.46–0.71; *P* < 0.00001). Also, EESs were significantly more effective in the reduction of TVR (Figure 3(c)). The incidence of TVR at all followups was 7.0% (294 of 4194) among patients treated with the EES and 9.7% (243 of 2517) among patients treated with the PES (OR: 0.64; CI: 0.54–0.77; *P* < 0.00001).

## 4. Interpretation

This meta-analysis clearly demonstrates that EES compared with PES reduced the incidence of early, late, and very late ST by about two-thirds. EES also reduced the incidence of MI and TVR compared to PES. We did not detect statistically significant difference in the rate of all cause death and cardiovascular mortality between EES and PES.

The finding of increased rates of late ST with first generation DES versus BMS has thought to be a major limitation of this technology [1]. It would be a significant clinical advance if new generations of DES are able to have comparable rates of late ST as bare stents with preserved benefits in terms of restenosis.

While individual randomized trials of EES versus PES showed differences in primarily early ST (within 30 days), the effect on late (31–365 days) and very late ST (>365 days) was uncertain. EES were developed to improve safety outcomes among patients including decreasing the risk of late and very late ST. Our meta-analysis showing differential rates of ST between EES and PES is important especially knowing how fatal ST can be and knowing that dual-antiplatelet therapy use has the risk of causing bleeding. Different durations of dual-antiplatelet therapy depending on the type of the stent used are being assessed [17, 18]. The dual-antiplatelet therapy study [17] which randomized patients with DES (different DES allowed) and BMS to different durations of dual antiplatelet therapy (12 versus 30 months) finished recruiting patients, and its results will help in defining the optimum duration of treatment that will weigh the risk against benefit of dual antiplatelet therapy. The risks and benefits of extending Clopidogrel duration may depend on the particular DES platform used.

The consistency of the reduction of ST in the individual trials suggests that this finding is true. The EES and PES group were well balanced for baseline characteristics, peri-procedural and postprocedural antiplatelets and anti-thrombotic drugs. It has been shown that the Xience V, EES has a more rapid rate of re-endothelialisation compared to PES. This finding may be related to the fact that the Xience V, EES release approximately 80% of its drug within 30 days and nearly all drug within 4 months [1]. Alternatively, the differential rates of ST may be related to differences in polymers, strut thickness, stent design, stent durability and the elution properties of Everolimus. Our knowledge about EES is rapidly expanding, and there are many questions yet to be answered. For example, a recent study comparing EES and PES found that in diabetic patients there were no significant differences in safety or efficacy outcome between both groups [19]. Finally, our meta-analysis demonstrated that TVR was reduced with EES versus PES (7.0% versus 9.7%; OR: 0.64; CI: 0.54–0.77; *P* < 0.00001) with the number needed to treat (NNT) of 37 patients. Regarding ST, we found that the NNT with EES compared with PES in order to prevent one case of ST is about 63 patients.

## 5. Limitations

The limitations of the current analysis are first, that individual patient data was not available, and second, the current dataset is underpowered for the outcome of mortality. However, the strengths of the analysis are consistent findings from the studies, and the large effect size with regards to 60–70% reduction in ST. Another limitation is that the results of the meta-analysis are only applicable to the XIENCE V platform and TAXUS Express and TAXUS Liberte, as these were the tested platforms in the randomized trials. Further studies will be needed for other stent platforms using Everolimus with different polymers and stent designs. Finally, this meta-analysis does not provide a comparison of EES versus BMS.

## 6. Conclusion

EES are superior to PES in terms of TVR and reduce the rates of early, late, and very late ST.

## Figures and Tables

**Table 1:** Description of the included trials.

Source	Number of patients		Inclusion criteria	Key exclusion criteria	Clinical follow-up duration, months
	EES	PES			
Kedhi et al. [6], 2010	897	903	Consecutive patients referred for elective or emergent PCI	Planned major surgery within 30 days	24

Garg et al. [5], 2009	223	77	Ischemia and vessel size 2.5–4.25 mm and lesion length ≤28 mm	Recent MI, LVEF ≤30%, left main, heavily calcified lesion, or visible thrombus	48

Stone et al. [7], 2009	669	332	Stable, unstable angina or inducible ischemia with vessel 2.5–3.75 mm diameter and lesion length ≤28 mm	Recent MI, LVEF <30%, LM bifurcation, by-pass graft, calcification, and thrombus	36

Stone et al. [8], 2010	2458	1229	Angina or ischemia with vessel 2.5–3.75 mm diameter and lesion length ≤28 mm	Recent MI, LVEF <30%, left main bifurcation, total occlusion, heavy calcification, total occlusion, restenosis, and visible thrombus, and vein graft PCI	24

Abbreviations: EES, Everolimus-Eluting Stent; PES, Paclitaxel-Eluting Stent; MI, Myocardial Infarction; LVEF, Left Ventricular Ejection Fraction; PCI, Percutaneous Coronary Intervention.

**Table 2:** 

Source	Stent platform used		Age, mean, y (SD)		Unstable anginaor NSTEMI (%)		STEMI (%)	Diabetes Mellitus (%)		Percentage of patients on dual antiplatelets at 1 year (%)	
	EES	PES	EES	PES	EES	PES		EES	PES	EES	PES
Kedhi et al. [6], 2010	XIENCE V	TAXUS Liberté	62.9 (55.4–71.1)	63.6 (55.7–72.9)	34	36	EES 27 PES 23	17.1	19	n/a	n/a

Garg et al. [5], 2009	XIENCE V	TAXUS Express or TAXUS Liberté	62.0 (10.0)	62.0 (9.0)	27	32	n/i	23	24	n/a	n/a

Stone et al. [7], 2009	XIENCE V	TAXUS EXPRESS2	63.2 (10.5)	62.8 (10.2)	18.7	25.1	n/i	29.6	27.9	71.7	71.7

Stone et al. [8], 2010	XIENCE V	TAXUS Express	63.3 (10.5)	63.3 (10.2)	27.7	28.9	n/i	32	32.5	91.9	92.2

Abbreviations: EES: everolimus-eluting stent; PES: paclitaxel-eluting stent; n/i: not included; n/a: not available; SD: standard deviation.
